# Correlation of surface-enhanced Raman spectroscopic fingerprints of kidney transplant recipient urine with kidney function parameters

**DOI:** 10.1038/s41598-021-82113-7

**Published:** 2021-01-28

**Authors:** Zhongli Huang, Shijian Feng, Qiunong Guan, Tao Lin, Jianhua Zhao, Christopher Y. C. Nguan, Haishan Zeng, David Harriman, Hong Li, Caigan Du

**Affiliations:** 1 Department of Urology, Institute of Urology, Organ Transplantation Center, West China Hospital, Sichuan University, 37 Guoxuexiang, Chengdu, 610041 China; 2 Department of Urologic Sciences, University of British Columbia, 2660 Oak Street, Vancouver, BC V6H 3Z6 Canada; 3 Imaging Unit, Integrative Oncology Department, BC Cancer Research Center, 675 W 10th Ave, Vancouver, BC V5Z 1L3 Canada

**Keywords:** Biological techniques, Biotechnology, Medical research, Neurology, Urology, Nanoscience and technology

## Abstract

Routine monitoring of kidney transplant function is required for the standard care in post-transplantation management, including frequent measurements of serum creatinine with or without kidney biopsy. However, the invasiveness of these methods with potential for clinically significant complications makes them less than ideal. The objective of this study was to develop a non-invasive tool to monitor the kidney transplant function by using Surface-Enhanced Raman Spectroscopy (SERS). Urine and blood samples were collected from kidney transplant recipients after surgery. Silver nanoparticle-based SERS spectra of the urine were measured and evaluated using partial least squires (PLS) analysis. The SERS spectra were compared with conventional chemical markers of kidney transplant function to assess its predictive ability. A total of 110 kidney transplant recipients were included in this study. PLS results showed significant correlation with urine protein (*R*^2^ = 0.4660, p < 0.01), creatinine (*R*^2^ = 0.8106, p < 0.01), and urea (*R*^2^ = 0.7808, p < 0.01). Furthermore, the prediction of the blood markers of kidney transplant function using the urine SERS spectra was indicated by *R*^2^ = 0.7628 (p < 0.01) for serum creatinine and *R*^2^ = 0.6539 (p < 0.01) for blood urea nitrogen. This preliminary study suggested that the urine SERS spectral analysis could be used as a convenient method for rapid assessment of kidney transplant function.

## Introduction

Kidney transplantation represents a gold standard treatment for patients with end-stage renal disease (ESRD)^1^. Donor and recipient factors, surgical as well as immunologic factors may all contribute or cause the impaired graft function either immediately following the transplantation operation, termed delayed graft function, or at any other time points after transplantation^2–5^. Allograft dysfunction is typically revealed by serial laboratory tests showing rising serum creatinine (SCr), along with other potential findings including decreased urine volume, and an increase in blood pressure, pyuria and proteinuria^6^. Acute rejection (AR), acute tubular necrosis (ATN) and cyclosporine/tacrolimus toxicity are some of the more common etiologies of kidney transplant dysfunction^7–9^. If these issues are not properly identified and addressed, they may lead to permanent deterioration of kidney transplant function. Thus, patients with kidney transplants need routine monitoring of their kidney transplant function as a part of post-transplantation management^10–12^.

Conventional methods for evaluating the functional status of transplanted kidneys include clinical assessment of the patients based on volume status and urine output, serial SCr measurements with estimated glomerular filtration rates (eGFR), and even isotope-based examinations in rare circumstances^12,13^. If AR is suspected, renal transplant ultrasounds are often performed followed by transplant biopsy to confirm a pathologic diagnosis for decision of the treatment plan. The procedure of the biopsy can be uncomfortable for patients and can lead to clinically significant complications, such as bleeding or allograft loss in the most extreme cases. Therefore, a non-invasive clinical tool which accurately determines the kidney transplant function is definitely desirable from both a patient and clinician perspective.

Raman spectroscopy (RS) is a specialized and non-invasive tool that emits an incident laser light though a fluid sample, resulting in scattered photons that produce a shift in wavelength dependent on the chemical composition of the fluid^14–17^. The intensity of Raman scattering is proportional to the magnitude of the change in molecular polarization. Previously, the application of RS was limited because of its low sensitivity, high costs and lack of accessible, on-site analysis^18^. Recent technological advance of RS has brought forth a readily available, portable and relatively cheap RS device^19^. Surface-enhanced Raman Spectroscopy (SERS) is a subtype of RS, designed specifically to increase its sensitivity of low concentration analytes, in which the treatment of a sample with an enhancement material significantly amplifies the electromagnetic fields of adsorbate molecules that are generated by the excitation of localized surface plasmons (LSP)^20^. Several SERS enhancement materials have been described (e.g. colloidal metals and roughened metals, including gold, silver and copper). These metal enhancers can increase the intensity of the Raman signal up to 10^4^ to 10^6^ fold^21^. Our previous study demonstrated that our home-made colloidal silver nanoparticles (Ag NP) are a reliable and reproducible enhancement material for SERS^22^. The objective of the current study was to evaluate the feasibility of using Ag NP-based SERS of patients’ urine samples to determine the kidney transplant function as compared to its conventional biochemical markers.

## Method and materials

### Specimen and data collection

Both urine and blood samples were collected from kidney transplant patients in the Department of Urology of West China Hospital in Sichuan University. These samples were collected concurrently within 3 days of the kidney transplant surgery. As following a standard operating procedure, the blood samples were sent to the clinical biochemistry laboratory in the West China Hospital for SCr and blood urea nitrogen (BUN) determination. The urine samples were collected by the patients themselves by using a 50 mL sterile tube. The urine samples were then delivered to the hospital laboratory within 8 h after collection. The urine tubes were centrifuged at 1500×*g* for 10 min to remove the cells and debris, and only the resultant supernatants were stored in aliquots at − 80 °C refrigerator prior to use. The levels of protein and creatinine in the urine samples were determined in the Clinical Chemistry Laboratory at Vancouver General Hospital (Vancouver, British Columbia, Canada).

### Silver (Ag) nanoparticles and urine sample preparation

Ag colloids were prepared using hydroxylamine hydrochloride and Ag nitrate as described previously^23,24^. Briefly, the glassware used in the following procedures was cleaned with chromic acid lotion, and then rinsed thoroughly with milli-Q water before use. 4.5 mL of 0.1 M NaOH solution was rapidly added to 5 mL of 6 × 10^−2^ M hydroxylamine hydrochloride solution, and the mixture was stirred until a homogeneous suspension was formed. The resultant solution was mixed with 90 mL of 1.11 × 10^−3^ M AgNO_3_ aqueous solution at room temperature until the presence of a milky-grey color. This Ag colloid suspension was then stored in the dark before use. In order to ensure consistent shape and size of the Ag NP, all the experimental steps and processes were standardized. Once ready for use, the Ag colloids were pelleted by centrifugation at 10,000×*g* for 10 min. A transmission electron microscopy (TEM) image of Ag nanoparticles was taken with the sizes of the synthesized Ag NP follow a normal distribution with a mean diameter of 35 ± 5 nm (Suppl. Figure 1). The pelleted Ag NP were then mixed with urine samples at a 1:1 ratio in a rectangle aluminum plate (10 µL total volume). The same batch of Ag NP was used for one round of SERS measurement of all of urine samples at the same time to avoid the probable inconsistency of the Ag NP. The repeated measurements (technical replicates) of the same samples were performed using the same or a different batch of the Ag NP. The Ag colloid/urine mixture was then air-dried at room temperature for 60 min prior to SERS measurement to facilitate molecular aggregation. If the prepared samples displayed the so called “coffee ring effect” or overt crystal formation (few cases), they were re-made until the absence of this problem. Of note, aggregation agents are believed to increase the signal intensity of SERS, however we elected to forego use of such agents in hopes of streamlining the sample preparation process.

### SERS measurement

SERS spectra of the Ag colloid/urine mixture were measured with a commercial Raman spectrometer (AURA, Verisante Technology, Vancouver, BC, Canada) equipped with a 785 nm diode laser for Raman excitation, similar to the prototype reported previously by Zhao et al.^25,26^. The laser beam was delivered to the sample through a single fiber with a core diameter of 200 µm. Raw signals were collected by a probe consisting of a central excitation fiber surrounded by one hundred and ten 100-µm core-diameter collection fibers, and then delivered to the spectrometer. Each SERS spectrum was acquired with 150 mW excitation laser power and an integration time of one second with a spectral resolution of 8 cm^−1^ in the wavenumber range of 500–1800 cm^−1^. For each dried droplet or sample, three spectra were measured with spotting the whole sample area on the aluminum slide, and the mean was used in subsequent data analysis. A monitor was also used to minimize the heterogeneity of each test of the same sample by visualizing the spectrum simultaneously during the measurement. If the visualized spectrum was largely different from each other, another 3–6 spectra were then obtained until a consistency was achieved.

### Data analysis

The resultant SERS spectrum of the urine samples in the wavelength range of 500–1800^–1^ cm were extracted by subtracting the fluorescence background using the fifth-order polynomial fitting algorithm^27^. Partial least squares (PLS) regression with leave-one-out cross-validation (LOO-CV) of the whole spectra was used to quantify the biochemical parameters of kidney transplant function including urine protein, urine creatinine (UCr), urine urea, SCr and BUN. The concentrations of these parameters obtained from blood/urine biochemistry analysis was used as a gold standard to compare the SERS urine analysis. TIBCO STATISTICA software (Version 10, TIBCO SOFTWARE Inc., Palo Alto, CA, USA) was used for PLS regression analysis.

### Ethical approval

This study (Approval Number: 2019(748)) was approved by the ethics committee at West China Hospital of Sichuan University, and all participants signed the informed consent prior to sample collection. Sichuan University and West China Hospital ethics committee confirmed that all the research activities were performed in accordance with relevant guidelines and regulations.

## Results

A total of 110 kidney transplant recipients (80 male and 30 female; mean age of cohort 41.5 years) participated in this study. The urine biochemistry results revealed that the range of urine protein was 0.05–6.83 g/L, UCr 1.03–22.49 mmol/L and urine urea 29.6–535.8 mmol/L. The biochemical results of blood tests indicated that the range of SCr was 39–1461 µmol/L and BUN 3.2–25.1 mmol/L. The demographic characteristics of patients and their urine and blood test results were summarized in Table 1.

**Table 1:** Basic characteristics of kidney transplant recipients.

Category	Ratio/range	Normal range
Gender (male/female)	80/30	–
Age (average, quartile) (years)	14–66 (41.5, 31–53.5)	–
Urine protein (g/L)	0.05–6.83	< 0.03
Urine creatinine (mmol/L)	1.03–22.49	–
Urine urea (mmol/L)	29.6–535.8	–
Serum creatinine (μmol/L)	39–1461	37–110
Serum BUN (mmol/L)	3.2–25.1	3.13–8.17

**Figure 1:**
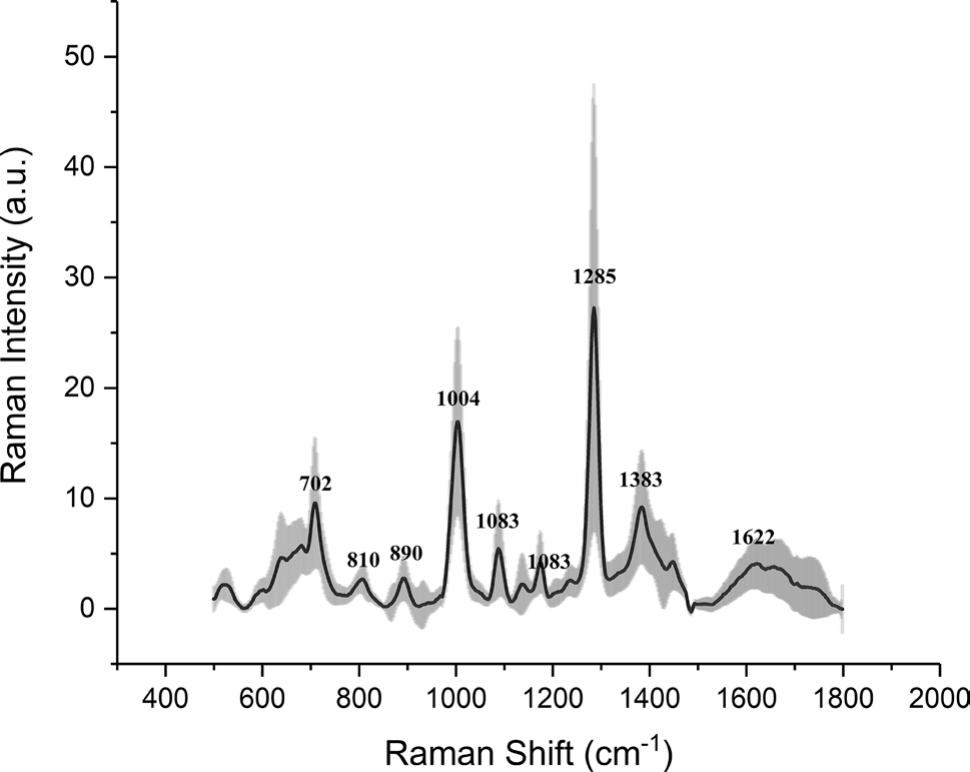
The SERS spectra of patients’ urine samples. The main SERS peaks (vibrational assignments) of tested urine samples were assigned to 1383, 1285, 1004, and 702 cm^−1^, which represent for CH3, urea, cholesterol, and cytosine band.

**Figure 2:**
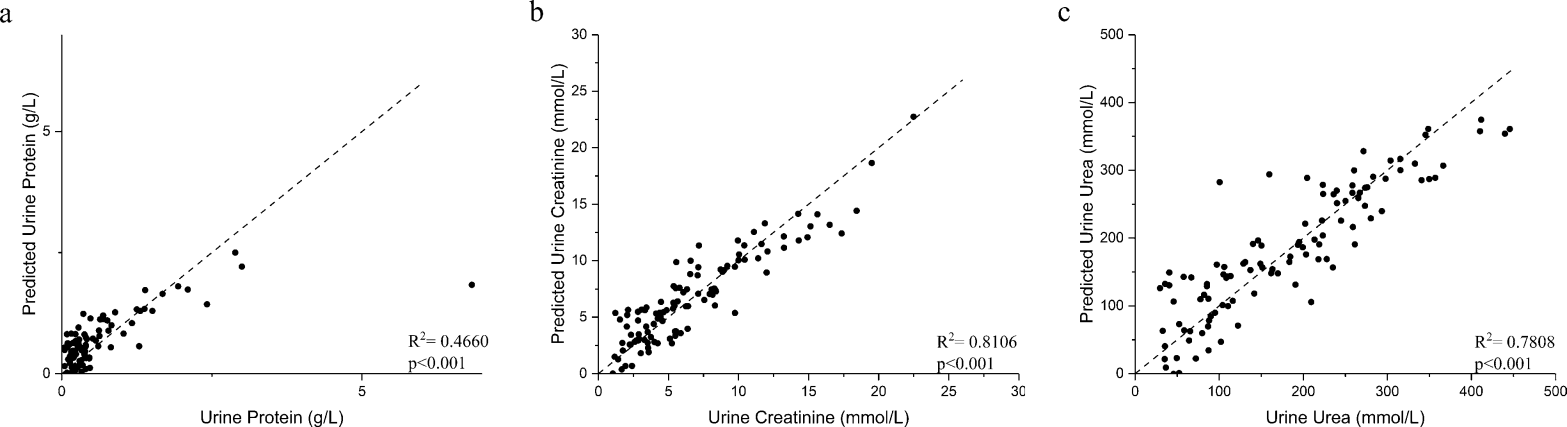
The PLS analysis generated models were used to predict concentrations of molecules in the urine. PLS analysis for urine protein (**a**), UCr (**b**), and urine urea (**c**). The results demonstrated that R^2^ = 0.4660 for urine protein (**a**) (p < 0.001), R^2^ = 0.8106 for UCr (**b**) (p < 0.001), R^2^ = 0.7808 for urine urea (**c**) (p < 0.001).

**Figure 3:**
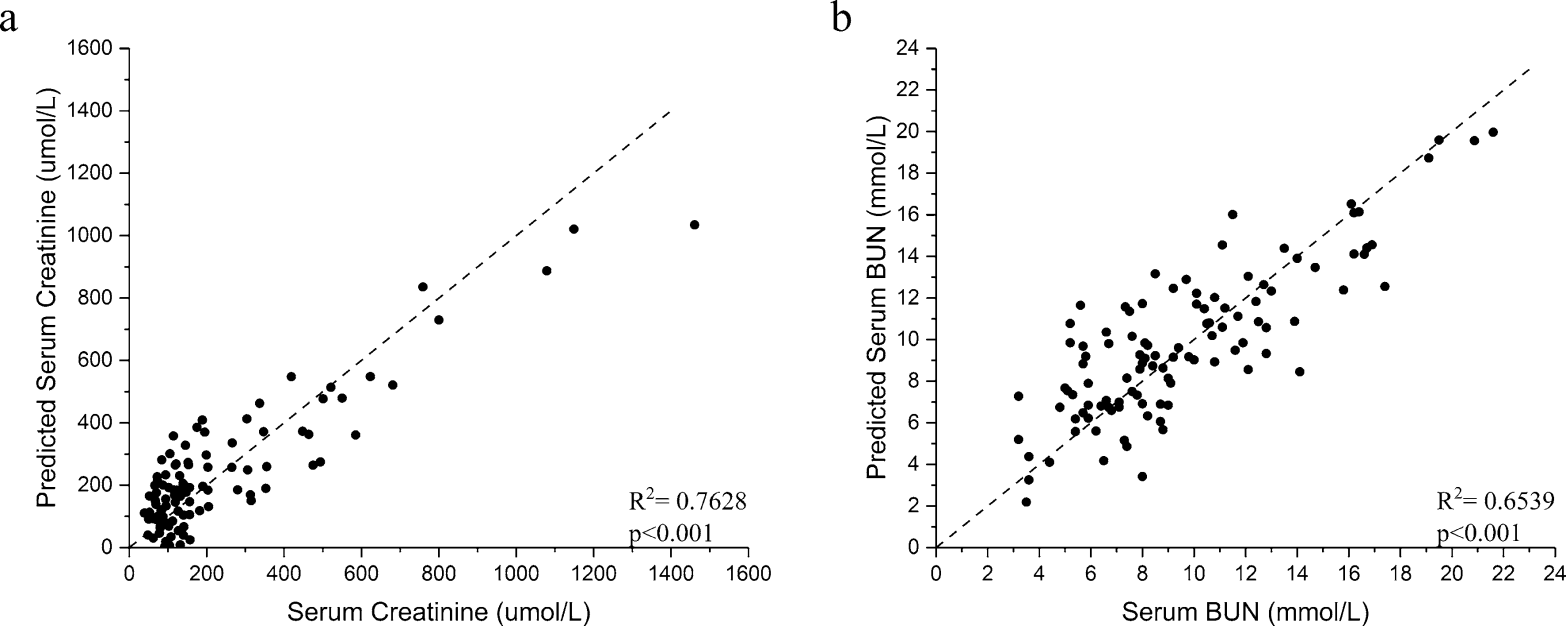
The PLS analysis generated models were used to predict the concentrations of blood biomarkers for kidney transplant function. The results showed that R^2^ = 0.7628 for SCr (**a**) (p < 0.001) and R^2^ = 0.6539 for BUN (**b**) (p < 0.001), respectively.

The SERS spectra of Ag NP/ urine mixtures were measured under the same conditions (1 day after mixture preparation). The SERS spectra of samples revealed many dominant vibration bands, implying a tight interaction between the Ag colloid and urine molecules resulting in the signal enhancement. As shown in Fig. 1, the main SERS peaks (vibrational assignments) of urines were assigned to 1285, 1004, 702, and 1383 cm^−1^, which biochemically corresponds to cytosine, urea, cholesterol, and CH3 band, respectively^28^. Other biochemical assignments of the SERS peaks showed in Fig. 1 are summarized in Table 2. To explore if the SERS could be used as a non-invasive tool to assess the status of kidney transplants, PLS generated models were utilized to predict the concentrations of kidney transplant function markers in the urine and blood. Figure 2 showed the results of PLS analysis of the urine protein, UCr, and urine urea as compared to the SERS spectra, respectively. As a reference for the prediction ability of PLS analysis, *R*^2^ > 0.67 indicated the high predictive accuracy, *R*^2^ range of 0.33–0.67 the moderate predictive accuracy, *R*^2^ range of 0.19–0.33 the low predictive accuracy and *R*^2^ < 0.19 unacceptable variables^29^. The urine results revealed an *R*^2^ = 0.4660 (moderate) for urine protein (*p* < 0.001), *R*^2^ = 0.8106 (high) for UCr (*p* < 0.001), and *R*^2^ = 0.7808 (high) for urine urea (*p* < 0.001). Figure 3 showed the results of PLS analysis of the blood SCr and BUN as compared to the urinary SERS spectra, respectively. The blood results revealed an *R*^2^ = 0.7628 (high) for SCr (*p* < 0.001) and *R*^2^ = 0.6539 (moderate to high) for BUN (*p* < 0.001). Because the low concentrations of kidney transplant function biomarkers—normal transplant function are not a concern in clinical care, we calculated the standard deviation (SD) of their high concentrations according to the borderline level. For the urine protein < 0.3 g/L, the calculated SD was 0.33 g/L, and > 0.3 g/L, the SD 0.44 g/L. For the UCr < 7 mmol/L, the calculated SD was 1.69 mmol/L, and > 7 mmol/L, the SD 1.64 mmol/L. For the urine urea < 266 mmol/L, the SD was 47.88 mmol/L, and > 266 mmol/L, the SD 29.57 mmol/L. The serum biomarkers are mainly used for the kidney transplant function in the clinical practice. For the SCr < 110 mmol/L, the SD was 87.98 mmol/L, and > 110 mmol/L, the SD 108.21 mmol/L. For the BUN < 7.7 mmol/L, the SD was 2.15, and > 7.7 mmol/L, the SD 2.32 mmol/L.

**Table 2:** Summarize previous reported SERS peak positions and vibration mode assignments^1^.

Peak position (cm^−1^)	Major assignments
702	Cholesterol, cholesterol ester
810	Phosphodiester (Z-marker)
890	Protein bands
1004	Urea
1083	C–N stretching of proteins
1173	Cytosine/guanine
1285	Cytosine
1383	CH3 band
1622	Tryptophan (IgG)

## Discussion

In clinics, post-kidney transplant function is mainly determined by monitoring the level of SCr and calculating the eGFR. This involves needle-obtained blood specimens, which can be bothersome and even traumatize some patients. There are other methods to evaluate the renal function, such as 24-h UCr clearance and nuclear medicine scans. However, these methods are rarely performed for the post-transplant care secondary to multiple factors, including logistics, expense and concerns regarding the inaccuracy when the kidney transplant function is poor. Therefore, a rapid and non-invasive tool to monitor the kidney transplant function would be a desirable alternative to these conventional means. Nowadays, non-invasive imaging methods including ultrasonography (US), computed tomography (CT), magnetic resonance imaging (MRI), and renal scintigraphy (RSG) are commonly used in clinics; however, these imaging methods have disadvantages over one or the other. US has a high inter-observer variability that makes difficulties in imaging interpretation. CT and MRI can provide the details of renal transplant anatomy and the surrounding tissue, but it is hard to measure the kidney function using these techniques. RSG, on the other hand, can shows the excretion patterns, leakage, and morphology of the kidney, it however cannot provide necessary information to differentiate between AR and ATN. Furthermore, the most concern about the nuclide seriation is low discharge rate due to the poor kidney transplant function.

Urine, a product of the kidney, contains thousands of molecules that reflect the body’s homeostasis and metabolic state at any given time. Also, it represents a specimen that is readily available via non-invasive means. Of most significance for renal function and kidney health can be determined by the urine protein, UCr and urine urea measurements. For example, healthy kidneys do not allow much protein to pass through the glomerular filters, but a damaged or diseased kidney may leak a large amount of the proteins, such as albumin, from the blood into the urine. Thus, it is reasonable to assess the kidney transplant function through the urine chemical measurement.

In the present study, we used SERS as a non-invasive tool to assess the kidney transplant function after surgery. Our data potentially demonstrated the feasibility of using SERS to monitor the kidney transplant function in the early post-operative period. We found a strong correlation between SERS spectra and biochemical substances in both the urine and the blood (urine protein/UCr/urea, SCr/BUN) (p < 0.01). Previously, we demonstrated that SERS spectra are able to detect or predict the renal damage in a rat model^24^. It has been acknowledged that the chemical composition of the urine from kidney transplant recipients is more complicated than those from the rat models and non-kidney transplant patients (e.g. kidney disease patients), as transplant patients have some remnant kidney function and take immunosuppressants routinely (e.g.. tacrolimus, mycophenolate mofetil, etc.). The different therapeutic drugs and the activity of the remnant kidney might result in different urine composition from one patients to the other. In the present study, we successfully showed that the urine SERS spectra of a small cohort of the kidney transplant recipients are capable of predicting essential kidney biomarkers in the presence of the interference from these uncertain (remnant kidney function and therapeutic drugs) and other unknown factors. There are other studies in literature have also have assessed the abilities of RS to detect the biochemical constituent concentrations in the urine. For example, the creatinine in unaltered human urine from a calibration dataset using RS is indicated by an Root Mean Square Error (RMSE) of 0.4332 mmol/L^30^. In the measurement of 61 human urine samples (excluding 12 outliers) using liquid-core optical fiber-based RS and multivariate statistical analysis, and the statistical error for creatinine quantification is 0.4508 mmol/L^31^. In Dou et al.’s study, the correlation coefficients between the concentrations of the urea and creatinine in the urine samples are presented by the intensity of the Raman peaks at 1013 and 692 cm^−1^ in RS, which show a *R*^2^ = 0.991 and *R*^2^ = 0.998, respectively. And the detection limits in this study are 174.93 and 13.2603 mmol/L, respectively^32^. Wang et al. have also investigated the SERS for the measurement of the concentration of creatinine in both artificial and human urine samples, demonstrating a correlation coefficient of r = 0.99 in the artificial urine samples over the range of 3.3946–13.6139 mmol/L, and r = 0.96 in the human urine samples over the range of 0.2263–10.1662 mmol/L^33^. In a follow-up study, these authors have reported *R*^2^ = 0.968 in a linear correlation of creatinine concentrations with the range from 0.442 to 15.1167 mmol/L^34^. Saatkamp et al. report the partial selected RS spectra to predict the urine urea and UCr concentrations with the correlation coefficient of r = 0.90 and r = 0.91, respectively^35^. In a separated study, they have identified the peaks specifically for the creatinine with R^2^ = 0.968 by using creatinine spiked in three different solutions: creatinine in water, mixture of creatinine and urea in water, and creatinine in artificial urine within physiologically relevant concentrations^36^. All these results may support the concept that SERS may be considered to be a reliable technique for monitoring the functions of transplanted kidneys non-invasively and rapidly.

As compared to conventional methods, SERS has many advantages. First, it is non-invasive. By using an optical instrument, it does not cause any damage to the patient’s body and the specimens can be non-invasively and conveniently obtained. In clinic, one of the severe complications after surgery is infection^37^. This is especially true for those transplant patients who regularly take immunosuppressant drugs, to whom the rate of infection and mortality are higher than the surgical patients without transplants^38^. To minimize the infection rates, a non-invasive method is required and should be the best choice. Moreover, for kidney transplant patients, a routine monitoring of the transplant function after the surgery is very critical for prolonging the survival of the transplants. The results from the present study indicate that SERS may provide a convenient, non-invasive and pain-free option for routine check-up of those patients. Second, SERS can detect the change of multiple substances in one sample quickly. Conventional biochemical methods such as enzyme-linked immunoreactivity method only examine one substance at a time, which is time-consuming. In technology, SERS is designed to detect the covalent bond inside the molecules which is different among different molecules. Thus, it is capable of differentiate the multiple substance at the same time. Third, potentially SERS is a more convenient, cheaper (cost-effective) and faster tool than conventional techniques in clinical biochemical laboratories. The SERS procedure can be completed as short as 1 s in the instrument, and the data can be calculated and reported immediately. The last but the least, it has great potential in other fields, such as rapid assessment of the deceased kidney donors where the baseline renal function can sometimes be in question.

One has to acknowledge the limitation of this preliminary study, which was mainly related to the limited number of patients (110 patients) from a single transplant center. In this small cohort, the distribution of some variables such as urine protein was not such large, the ability of Ag NP-based SERS was low in the prediction of the urine protein and perhaps the SCr.

## Conclusion

In this study, we have demonstrated the highly feasibility of using non-invasive Ag NP-based SERS of the urine to predict the kidney transplant function, indicated by the fact that the urine spectra of SERS are predictive of both the urine and blood biochemical constituents (e.g. urine protein, UCr and urine urea, and SCr and BUN) that conventionally reflect the health of the kidney. Further researches are warranted, ideally in a larger cohort, to validate the findings from this study and to build more precise models for monitoring the kidney transplant function in patients in future.

## Supplementary Information

Supplementary Figure 1.

## Data Availability

The data supporting the findings of this study was included in this manuscript, and the original files are available from the corresponding author upon reasonable request.

## Abbreviations

Ag NP: Silver nanoparticles
AR: Acute rejection
ATN: Acute tubular necrosis
BUN: Blood urea nitrogen
CT: Computed tomography
eGFR: Estimated glomerular filtration rates
ESRD: End-stage renal disease
LSP: Localized surface plasmons
MRI: Magnetic resonance imaging
PLS: Partial least squires
RS: Raman spectroscopy
RSG: Renal scintigraphy
SCr: Serum creatinine
SD: Standard deviation
SERS: Surface-enhanced Raman spectroscopy
TEM: Transmission electron microscopy
UCr: Urine creatinine
US: Ultrasonography
