# Supplementary material for: Correlation of surface-enhanced Raman spectroscopic fingerprints of kidney transplant recipient urine with kidney function parameters
Source: Sci Rep. 2021 Jan 28;11:2463. doi: 10.1038/s41598-021-82113-7 (PMC7843595; doi:10.1038/s41598-021-82113-7)

Correlation of Surface-Enhanced Raman Spectroscopic Fingerprints of Kidney Transplant Recipient Urine with Kidney Function Parameters

Running Title: Surface-Enhanced Raman Spectroscopy and Kidney Transplantation

Shijian Feng[1,2#], Zhongli Huang[1,2#], Qiunong Guan[2], Tao Lin[1], Jianhua Zhao[3], Christopher YC Nguan[2], Haishan Zeng[3], David Harriman[2], Hong Li[1, *], Caigan Du[2,*]

Suppl. Fig. 1.

Transmission electron microscopy (TEM) image of the prepared Ag nanoparticles. The sizes of particle follow a normal distribution with a mean diameter of 35 nm and standard deviation of 5 nm.

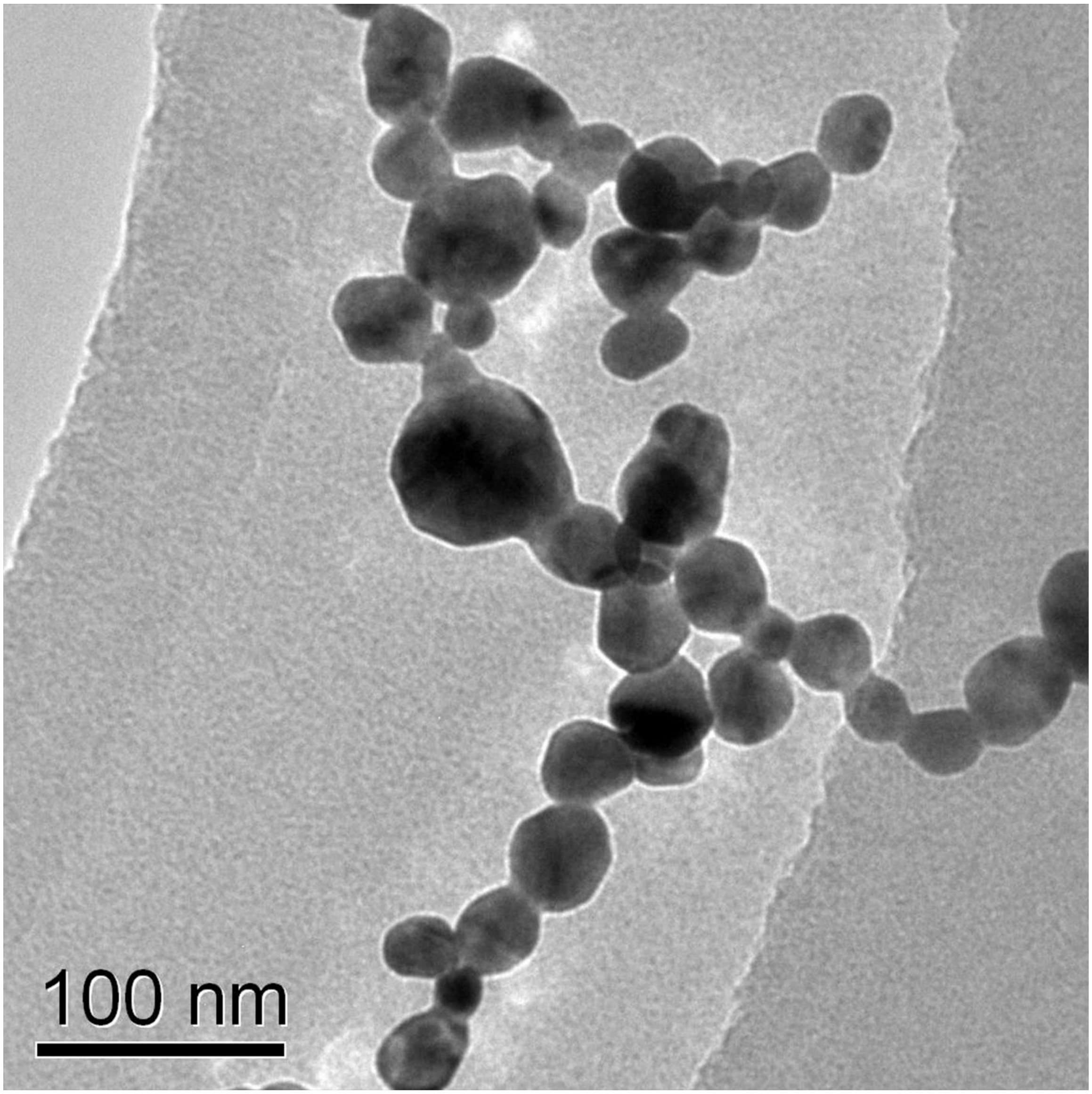

Supplement: Supplementary file 1 — Supplementary Figure 1. [file 41598_2021_82113_MOESM1_ESM.pdf]
